# The Role of the Complement in Clear Cell Renal Carcinoma (ccRCC)—What Future Prospects Are There for Its Use in Clinical Practice?

**DOI:** 10.3390/cancers16030490

**Published:** 2024-01-23

**Authors:** Martina Panebianco, Chiara Ciccarese, Alessandro Strusi, Viria Beccia, Carmine Carbone, Antonio Agostini, Geny Piro, Giampaolo Tortora, Roberto Iacovelli

**Affiliations:** 1 Medical Oncology, Department of Medical and Surgical Sciences, Fondazione Policlinico Universitario Agostino Gemelli IRCCS, 00168 Rome, Italy; martina.panebianco@aslroma4.it (M.P.); chiara.ciccarese@policlinicogemelli.it (C.C.); carmine.carbone@policlinicogemelli.it (C.C.); agoan27@gmail.com (A.A.); geny.piro@policlinicogemelli.it (G.P.); giampaolo.tortora@policlinicogemelli.it (G.T.); 2 Medical Oncology, Department of Translational Medicine and Surgery, Catholic University of the Sacred Heart, 00168 Rome, Italy; ale.strusi94@gmail.com (A.S.); viria.beccia@gmail.com (V.B.)

**Keywords:** mRCC, complement, classical pathway, “aggressive complement” tumor, biomarkers, immunotherapy

## Abstract

**Simple Summary:**

In recent years the first-line treatment of advanced renal cancer cell was implemented by new combination strategy. However, despite numerous research efforts, the choice of the type of treatment is still entrusted to clinical parameters. In this review we deepened the role of the complement system (CS) as a prognostic and predictive marker in kidney cancer. In particular, we described the physiology of the CS, its interaction with the tumor microenvironment and its role in oncogenesis and tumor progression. Based on the data reported in the literature, we concluded that the CS has a negative prognostic role in this pathology and its predictive role of response to immune-checkpoint inhibitors should be tested in prospective studies in order to optimize personalization of treatment for our patients, with the aim of reducing toxicity.

**Abstract:**

In recent years, the first-line available therapeutic options for metastatic renal cell carcinoma (mRCC) have radically changed with the introduction into clinical practice of new immune checkpoint inhibitor (ICI)-based combinations. Many efforts are focusing on identifying novel prognostic and predictive markers in this setting. The complement system (CS) plays a central role in promoting the growth and progression of mRCC. In particular, mRCC has been defined as an “aggressive complement tumor”, which encompasses a group of malignancies with poor prognosie and highly expressed complement components. Several preclinical and retrospective studies have demonstrated the negative prognostic role of the complement in mRCC; however, there is little evidence on its possible role as a predictor of the response to ICIs. The purpose of this review is to explore more deeply the physio-pathological role of the complement in the development of RCC and its possible future use in clinical practice as a prognostic and predictive factor.

## 1. Introduction

In recent years, we have witnessed a change in the implementation of first-line treatment options for metastatic renal cell carcinoma (mRCC), with the introduction into clinical practice of two different immune checkpoint inhibitor (ICI)-based combinations, not directly compared to each other: the dual ICI regimen of ipilimumab + nivolumab (Ipi/Nivo) [1] or combinations of an ICI plus a VEGFR tyrosine kinase inhibitor (ICI + TKI) [2,3,4,5]. All the pivotal studies leading to the approval of ICI-based combinations stratified patients according to the International Metastatic RCC Database Consortium (IMDC) prognostic risk score, which relies only on clinical features [6]. Therefore, one of the main goals of the translational research aims at identifying new prognostic and predictive biomarkers of mRCC in tumor tissue, in order to select at diagnosis the patients who would benefit more from ICI rather than TKI. The identification of new biomarkers is essential for a better characterization of this disease and a tailored treatment selection [7,8,9,10,11,12,13]. One of the aspects that research has deepened in terms of RCC is the role of the complement system (CS) as a modulator of the tumor microenvironment (TME) and a possible prognostic and predictive factor in RCC. In fact, there is considerable evidence that the CS could play a key role in RCC progression and metastatization [14,15]. Roumenia et al. identified RCC as part of the so-called “aggressive complement tumors”, which are characterized by a high expression of the complement components of the classical and alternative pathways and are associated with a worse prognosis [16]. Moreover, some preclinical studies evaluated the predictive role of the CS in mRCC as an immune-sensitive marker [17,18].

The purpose of this review is to elucidate the role of the complement in the TME, particularly in RCC, and to evaluate its possible use as a prognostic and predictive factor of response to ICI-based combinations.

## 2. The Physiology of the Complement System

The complement was first discovered in 1996 by Jules Bordet. The scientist did not observe bacterial lysis when serum containing antibodies against bacteria, preheated to a temperature of 56 °C, was incubated with the pathogens. Therefore, since it was already known that antibodies are heat-resistant, he hypothesized the presence of an additional heat-labile serum component that could assist the action of the antibody-mediated lysis, which was named the “complement” [19]. The complement system (CS) involves about 50 constituents, such as pattern recognition molecules (PRM), protein components, proteases, regulators, and cell surface receptors [20], which are produced mostly by the liver [21]. At the beginning, the CS was considered part of innate immunity due to the important role it plays in opsonization, chemotaxis, the lysis of pathogens, and inflammation. However, new evidence shows that the CS acts as a bridge between our innate and adaptive immunity, as it is able to increase the antibody response, promote the T cell response, eliminate self-reactive B cells, and enhance immunologic memory. In addition, the CS maintains cell homeostasis by eliminating cellular debris and immune complexes [22]. The activation of the complement occurs according to a mechanism called a “cascade” with sequential activation of the various components that circulate in inactive form (zymogens). The CS can be activated in canonical and non-canonical manners [14].

### 2.1. Canonical Pathways of Complement Activation

**Figure 1:**
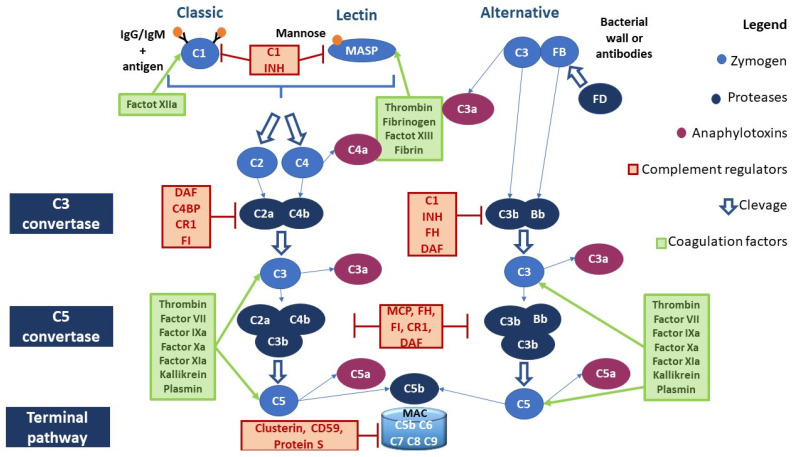
Canonical pathways of the complement activation and interaction of the coagulation system with the CS. Abbreviations. C1 INH, C1 inhibitor; DAF, decay-accelerating factor, or CD55, cluster of differentiation 55; C4BP, C4, binding protein; MCP, membrane cofactor protein, or CD46, cluster of differentiation 46; FH, factor H; FD, factor D; FI, factor I; MAC, membrane attack complex.

There are three canonical pathways of complement activation: classical, alternative, and lectin-driven. All three canonical pathways lead first to the formation of C3 convertase, which, in turn, cleaves C3 into two proteins (C3a and C3b). C3b is part of the C5 convertase that cleaves the C5 protein into C5a and C5b. C5b binds C6 and C7, forming the C5b,6,7 complex. C7, which is a hydrophobic protein, allows the insertion of the C5b,6,7 complex into the lipid double layer of the microbial membrane, where it becomes a receptor for C8 (complex C5b–8). The last step in the formation of the membrane attack complex (MAC) is the binding of the complex C5b–8 with C9. Then, the complex C5b–9 polymerizes and forms a pore in the bacterial membrane, which ultimately leads to the lysis of the cell (Figure 1).

#### 2.1.1. Classical Pathway

Classical pathway (CP) activation starts with the binding of the C1 fraction to the constant immunoglobulin domains IgG and IgM complexed to the antigen. The C1 fraction is a protein complex composed of C1q, C1r, and C1s subunits: C1q is assigned to the antibody binding, while C1r and C1s have protease activity. The binding of C1q to the immunoglobulins’ Fc region leads to the activation of C1r, which cleaves and activates C1s. C1s cleaves the next component of the complement cascade, the C4 fraction, generating C4a and C4b fragments: the first is released in the fluid phase, while the second will bind on the microbial surface. Another important component is the C2 fraction, which is cleaved by C1s after the binding with the C4b fragment, generating soluble C2b and C2a. C2a remains physically associated with the C4b fragment bound to the microbial surface. The complex C4b2a represents the C3 convertase of the CP [23] (Figure 1).

#### 2.1.2. Lectin Pathway

The lectin pathway (LP) is antibody-independent. It is activated by the interaction of microbial polysaccharides with circulating lectins such as mannose-binding lectin (MBL). MBL interacts with the associated serine protease (MASP), whose members are MASP-1, MASP-2, and MASP-3. MASP proteins have a homologous structure to that of the C1r and C1s proteases of the C1 fraction and perform very similar functions. The protein MASP-1 can form a tetrameric complex with MASP-2 similar to that formed by the subunits C1r and C1s; specifically, MASP-2 cleaves C4 and C2. The events resulting from this reaction are identical to those occurring in the classical pathway [24] (Figure 1).

#### 2.1.3. Alternative Pathway

The alternative pathway (AP) is activated by direct C3 binding to the bacterial wall or antibodies. C3 proteolysis forms C3b, which covalently binds to the microbial surface. This bond induces exposure to an additional binding site for factor B (FB). FB, which binds to C3b, is cleaved by a plasma serine protease called factor D (FD). The cleavage generates a small fragment called Ba and a large fragment called Bb that remains linked to C3b. The C3bBb complex represents the C3 convertase of the AP. C3b fragments, generated by the cleavage of C3, can bind to the surface of the microbe or bind to the C3 convertase itself, leading to the formation of a complex containing a Bb fragment and two C3b fragments (C5 convertase) [25] (Figure 1).

#### 2.1.4. Anaphylatoxins

C3a, C5a, and C4a represent the anaphylatoxins produced by the activation of the CS. C4a has been shown to play a possible role in the activity of macrophages and monocytes, but further studies are needed to discover the receptors and functions of this anaphylatoxin [26,27,28]. C3a and C5a exert their important inflammatory role through the C3aR and C5aR receptors expressed on the cells of the immune system, such as non-myeloid cells [29]. In particular, C3a and C5a have the following functions: (a) contribute to the activation of macrophages, eosinophils, and neutrophils by stimulating the production of reactive oxygen species (ROS); (b) promote chemotaxis; (c) stimulate the release of histamine from mast cells; (d) and promote vasodilation [29,30,31,32,33]. The role of the C5CL2 receptor, which binds both C5a and C3a, is still unclear [34].

Anaphylatoxins also play a fundamental role in adaptive immunity. The CR2 receptor, expressed by B cells, contributes to the activation of B-lymphocytes after interaction with C3d-opsonized pathogens [35,36,37]. C3a and C5a are able to modulate the differentiation of T cells through the production of specific cytokines from the T cells and antigen-presenting cells (APC) [37,38]. Furthermore, local C3 synthesis by the dendritic cells is necessary to induce a Th1 response [39].

### 2.2. Non-Canonical Pathways of Complement Activation

#### 2.2.1. Complement Activation through Coagulation Factors

Several studies have shown the possibility of activating the CS through proteases belonging to the coagulation system, and vice versa. Moreover, the CS may have both procoagulant (MAC) and anticoagulant action (C5a) [40] (Figure 1). For example, as reported in Table 1, factor XII is able to activate C1r and MASP1; thrombin leads to the activation of MASP1, MASP2, C3, and C4, whereas it inhibits the CS pathway by activating DAF [41,42,43,44,45]. Table 1 summarizes the interactions between the complement and coagulation systems.

#### 2.2.2. Local Complement Production

**Figure 2:**
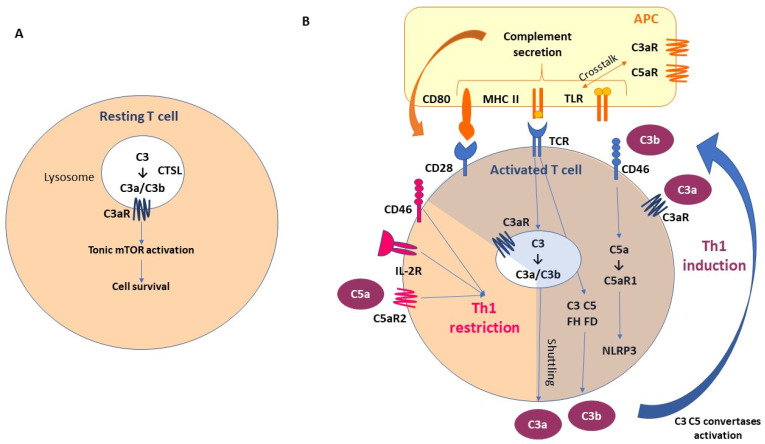
Local complement activation: the fundamental role of “complosome”. (**A**) T-cell-expressed protease cathepsin L (CTSL) processes C3 into biologically active C3a and C3b. “Tonic” intracellular C3a generation is required for homeostatic T cell survival through mTOR activation. This intracellular CS is defined as “complosome”. (**B**) As a result of T cell activation, the activated intracellular complement and complement receptors are secreted by T cells; C3a and C3b bind to C3aR and CD46 (membrane cofactor protein, MCP), respectively, and promote protective T Helper1 (Th1) differentiation. The activation of T cell receptors (TCRs) promotes also intracellular C3, C5, FH, FD secretion. Moreover, intracellular C5 activation (induced by CD46) and stimulation of C5a receptor 1 (C5aR1) promote pyrin-domain-containing protein 3 (NLRP3) inflammasome assembly, caspase-1-dependent interleukin-1β secretion, and induction of Th1 functional activation. In the early stages, CD46 promotes differentiation and expansion of T cells into Th1 effector lymphocytes. However, in the second phase, CD46, in association with the IL-2 receptor (IL-2R), leads to a Th1 restriction, promoting the secretion of IL-10. This is associated with the expression of C5aR2 via surface-shuttled C5a/C5a-desArg, which probably has a role in assisting this inhibitory signal. In addition, a synergistic effect has been observed between Toll-like receptor (TLR) and complement receptors expressed on the antigen-presenting cells (APCs). Abbreviations. APC, antigen-presenting cells; CTSL, T-cell-expressed protease cathepsin L; MHC II, major histocompatibility complex class II; TLR, Toll-like receptor; C3aR, C3a receptor; C5aR, C5a receptor; IL-2R, interleukin-2 receptor; NLRP3, NLR family pyrin-domain-containing 3.

Although the majority of complement components are produced by the liver, new evidence has suggested also the local production of C3 and C5. T cells are among the first discovered to be C3 producers. C3 is expressed in the lysosomes and the endoplasmic reticulum of resting T lymphocytes. T-cell-expressed protease cathepsin L (CTSL) processes C3 into biologically active C3a and C3b. “Tonic” intracellular C3a generation is required for homeostatic T cell survival through mTOR activation. This intracellular CS is defined as a “complosome” (Figure 2A).

As a result of T cell activation, the activated intracellular complement and complement receptors are secreted by the T cells; C3a and C3b bind to C3aR and CD46 (membrane cofactor protein, MCP), respectively, and promote protective T Helper1 (Th1) differentiation [27,59,60]. The activation of the T cell receptors (TCRs) promotes also intracellular C3, C5, FH, and FD secretion. Moreover, intracellular C5 activation (induced by CD46) and stimulation of C5a receptor 1 (C5aR1) promote pyrin-domain-containing protein 3 (NLRP3) inflammasome assembly, caspase-1-dependent interleukin-1β secretion, and the induction of Th1 functional activation [61,62]. In the early stages, CD46 promotes the differentiation and expansion of T cells into Th1 effector lymphocytes. However, in the second phase, CD46, in association with the IL-2 receptor (IL-2R), leads to a Th1 restriction, promoting the secretion of IL-10. This is associated with the expression of the C5aR2 via surface-shuttled C5a/C5a-desArg, which probably has a role in assisting this inhibitory signal [60,63]. In addition, a synergistic effect has been observed between Toll-like receptor (TLR) and the complement receptors expressed on the antigen-presenting cells (APCs) [64]. These mechanisms emphasize the importance of paracrine and autocrine complement secretion in the T-cell/APC interaction (Figure 2B), with the possibility of complement intracellular storage not being restricted to the immune cells.

### 2.3. Complement Negative Regulators

Several complement regulators (either soluble or membrane-bound) prevent aberrant activation of the CS (Figure 1). In particular, the negative regulators of the CS include:

(a) C1 inhibitor (C1 INH) is a competitive inhibitor of the C1r-C1s complex and MASP2 and interferes with the C3b–factor B interaction, blocking the activation of all three canonical pathways of CS;
(b) C3 and C5 convertase inhibitors: C4b-binding protein (C4BP) controls the activation at the C4 level of the CP and LP; factor H (FH) competes with FB to C3b-bond; complement receptor 1 (CR1) is capable of binding both C3b and C4b, displacing the link with Bb and C2b, and acts as a co-factor for factor I (FI), which degrades the fragment C3b; membrane cofactor protein (MCP) is capable of binding both C3b and C4b, displacing the link with Bb and C2b, respectively; and decay-accelerating factor (DAF) accelerates the decay of C3 convertases.
(c) MAC formation inhibitors: CD59 prevents the binding of the C9 fraction; S protein binds the C5b,6,7 complex, inhibiting insertion into the membranes; clusterin binds to C7 and the β-subunit of C8 and C9, inhibiting the correct complex assembly [65,66,67].

## 3. Cancer and Complement

The first evidence of an intrinsic interplay between the complement and cancer was demonstrated with the introduction of rituximab into lymphoma treatment. Rituximab is a chimeric (murine/human) monoclonal antibody that targets the CD20 receptor, expressed by the B cells. In particular, rituximab activates the classical pathway of the CS through the binding of its Fc portion to the C1q component. The activation of the complement results in the opsonization and lysis of cancer cells and in the recruitment of immune cells with phagocytic properties (i.e., macrophages and neutrophils) [14,68]. The complement has a dichotomous role in the tumor microenviroment (TME): in fact, the physiological complement concentrations activate the immune system’s response to cancer cells, whereas an aberrant and chronic activation of the CS has been related to an immunosuppressed TME [69]. Moreover, cancer cells evade the CS’s activity by hyper-expressing negative complement regulators such as CD35, CD46, CD55, and CD59 [70,71].

Complement proteins are produced in the TME by tumor-infiltrating immune cells and cancer cells and have access to the TME through vessels [72,73]. Complement activation is a response to tumor neoantigens and it has been shown to promote growth, progression, invasiveness, and tumor metastasization [74]. C5a is involved in the recruitment of myeloid-derived suppressor cells (MDSCs), resulting in suppressed antitumor immune responses [75]. The MDSCs inhibited the effector T cells, promoted angiogenesis, and were associated with an increased expression of immunomodulators such as programmed cell death 1 ligand 1 (PD-L1), arginase 1 (ARG1), cytotoxic T-lymphocyte antigen 4 (CTLA4), interleukin 10 (IL-10), IL-6, and lymphocyte-activation gene 3 (LAG3) in a murine model of lung cancer [76]. In addition, C5a promotes the extrusion of neutrophil extracellular traps (NETs), which are fibers of extracellular DNA released from the neutrophils. In particular, the nuclear protein high-mobility group box 1 (HMGB1) seems to be an endogenous stimulus produced by tumor cells that mediates the induction of NETosis in C5a-stimulated polymorphonuclear MDSCs (PMN-MDSCs). Furthermore, C5a facilitates the migration of these cells into the tumor, reducing the surface expression of β1 and β3 integrins and upregulating CD11b, a mediator of leukocyte extravasation [77].

Similar to C5a, C3a is involved in neutrophil recruitment [78]. Specifically, C3aR-dependent NETosis conduces coagulation and N2 polarization, promoting tumorigenesis. This shows a novel link between coagulation, neutrophilia, and complement activation [79]. Both C3a and C5a recruit tumor-associated macrophages (TAMs) and determine their differentiation into an M2-like phenotype, driving the immunosuppression of the T cells and tumor promotion [80,81]. M2 TAMs produce vascular endothelial growth factor (VEGF), fibroblast growth factor (FGF) chemokines, IL-17, IL-23, TGF-β, and other growth factors that determine the stimulation of vascular endothelial cell proliferation and the release of metalloproteases (MMPs), favoring tumor neovascularization, invasiveness, and progression [82,83,84].

**Figure 3:**
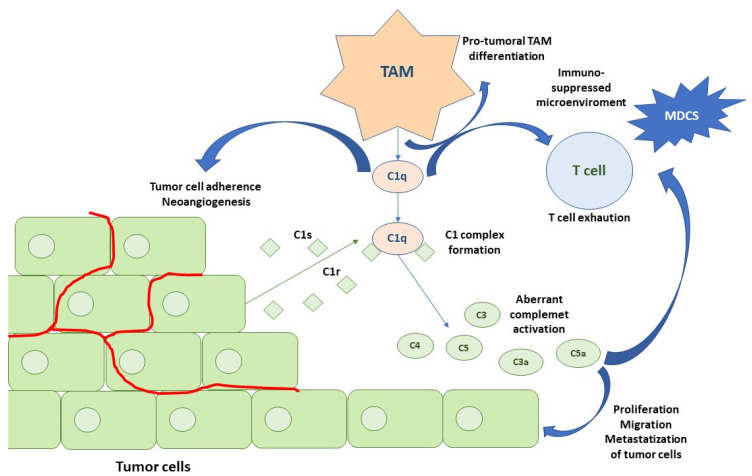
Activation of the CS in an RCC microenvironment. The first step is represented by C1q TAM secretion that contributes to pro-tumoral TAM phenotypes, T cell exhaustion, adhesion of tumor cells to the extracellular matrix, and neoangiogenesis. RCC cancer cells produce C1r and C1s, leading to an active C1 complex formation. This, in association with IgG deposits on tumor cells, promotes the classical pathway of the CS. Tumor cells also secrete the subsequent complement components, leading to C4 and C3 activation fragment deposition (C4b, C4d and C3b, iC3b, C3d). Moreover, anaphylatoxins C3a and C5a produced by the activation of the classical pathway modulate tumor cells and TME. Abbreviations: TAMs, tumor-associated macrophages; mDCs, myeloid dendritic cells.

A new C1q+ TAM subpopulation was recently identified in the TME. The chemokine CXCL-10, secreted by the C1Q+ TAM, activates and recruits CD8+ and CD4+ T cells, especially Th1 cells, binding to the receptor CXCR3 expressed by these immune cells. C1q+ TAM, favors T cell exhaustion. Moreover, C1q seems to perform the following functions: (a) activating an extracellular complement cascade and promoting chronic inflammation; (b) interacting with the endothelial cells promoting neoangiogenesis; (c) regulating macrophage polarization through autocrine action, interacting with LAIR1 (Figure 3).

Fibroblasts are also able to secrete C1q-containing exosomes and influence M2-like macrophage differentiation [85]. Autocrine stimulation of C3aR and C5aR1, expressed in the tumor cells, promotes tumor proliferation, triggering the phosphoinositide 3-kinase (PI3K)–AKT pathway [73]. Similarly, the C5b–C9 deposited on the cancer cells activates signal transduction pathways and induces cell cycle progression [86,87].

With regard to invasiveness and metastatization, both C3a and C5a play a role: C3a inhibits the expression of E-caderin, while C5a, through a C5aR1-mediated signal, stimulates the production of MMPs [88,89]. C3a activates the WNT–β-catenin pathway and enhances tumor cell proliferation, migration, and stemness in a mouse model with cutaneous squamous cell carcinoma [90].

Long pentraxin (PTX3) has been defined as a possible link between inflammation and cancer. PTX3 in physiological conditions acts as a functional ancestor of antibodies. It has an opsonic function via the Fc receptors, activates and regulates the complement cascade, regulates inflammation, and has a role in vascular biology [91,92,93,94,95,96]. Particularly, PTX3 binds C1q, activating the CP of the CS [93,96,97], modulates the LP by interacting with M-ficolins and MBL [98], and interacts with FH, modulating negatively AP activation through the recruitment of FH in PTX3-opsonized cells [99]. Therefore, PTX3 can be considered an oncosuppressor gene able to block complement-induced tumorigenesis. In fact, in knockout mice lacking the PTX3 gene, major susceptibility to mesenchymal and epithelial carcinogenesis; complement C3 deposition and higher C5a circulating levels, macrophage infiltration, cytokine production, and angiogenesis were observed. Therefore, this preclinical model suggests evidence of the central role of PTX3, as driven by complement activated macrophages, in counteracting tumor progression and of its loss potentially contributing to TME immunosuppression [100]. Moreover, loss of PTX3 was associated with genetic instability, contributing to malignant transformation [101].

## 4. The Prognostic Role of the Complement in Renal Cell Carcinoma

Based on the complement component gene expression analysis from The Cancer Genome Atlas (TCGA)’s database, four groups of neoplasms were characterized, including the so-called “aggressive complement” tumors. This group includes kidney renal clear cell carcinoma (KIRC), lung squamous carcinoma (LUSC), uveal melanoma (UVM), lower-grade glioma (LGG), and glioblastoma (GBM). The “aggressive complement” cancers highly express factors of both the classical and alternative pathways, which are significantly correlated with a worse prognosis [16]. Two important studies investigated the prognostic role of the C1q, C1s, C3, and C4 components in RCC [102,103]. Roumenia et al. conducted a study aimed at understanding the activation mechanisms of the CS in RCC and its impact on patients’ clinical outcome. This study included an analysis of cell culture, mouse models, three retrospective cohorts of patients (with a total of 303 patients), and a prospective cohort of 7 patients, affected by stages I–IV ccRCC. Immunohistochemistry analyses of the C1q expression were conducted. Firstly, tumors were scored into three groups according to the percentage of C1q non-neoplastic cells: score 0 (weak): <1%; score 1 (intermediate): 1–30%; and score 2 (strong): >30%. Later, given the worse prognosis of patients with a score of 2 compared to those with scores 0–1, all subsequent evaluations were performed separating tumors into C1q high (score 2) and C1q low (scores 0 to 1) staining. The same score was used for the histological coloration criteria of cytoplasmic C4/C3 and C4d/C3d deposits on the tumor cell membranes. The analysis was first performed on 106 patients with stages I–IV ccRCC (Cohort 1), which revealed that the expression of C1q was a significant negative prognostic factor in the overall population, but specifically in patients with advanced stage disease (III–IV stages) for both overall survival (OS) (*p* < 0.002) and progression-free survival (PFS) (*p* < 0.004). These findings were then validated using cohorts 2 and 3. Immunofluorescence analysis showed that macrophages were the major cell type producing C1q in ccRCC tumors (80% of the C1q+-infiltrating cells were CD68+CD163+ macrophages), whereas the tumor cells stained negative for cytoplasmic C1q. Moreover, the M5 macrophage subtype was the principal C1q productor, associated with exhausted T cells. In fact, a positive correlation was found between the C1q+ cell density and PD-1 (*p* = 0.012), as well as LAG3 (*p* = 0.0008) in the 102 patients of cohort 1. A correlation between the C1q gene expression and exhausted T cell markers was confirmed in ccRCC tumors in publicly available transcriptomic data from the TCGA database (*n* = 537). The co-localization of IgG deposits and C1q on the surface of the tumor cells (30% of cases) and the co-localization of C1q with C4d staining (about 50% of samples) demonstrated in situ CP activation in RCC. Patients with a high density of C4-producing tumor cells and high C4d deposits had a significantly worse OS (*p* = 0.007) and PFS (*p* = 0.013). C1q, C4, and C4d were independent prognostic factors in both univariate and multivariate analyses, as opposed to C3 and C3d. Moreover, the ablation of C1q, C4, and C3 in mice was associated with decreased tumor growth. Impaired vascularization and downregulated VEGF-C gene expression were observed in a C1q knockout murine model [102]. This study confirmed the intra-tumoral activation of the CS, thanks to the in loco production of C1q by the macrophages and given the tumor cells’ secretion of C3 and C4. Of note, C1q seemed to have a role in the angiogenesis and contributed to an immunosuppressed TME [102]. This study demonstrated for the first time the negative prognostic role of the complement components in RCC; moreover, it used cellular and animal models to reconstruct the intra-tumoral biological pathways through which complement activation occurs in RCC. This was fundamental to understanding the biological rationale behind the negative prognostic value of the CS, as well as its possible predictive role in identifying patients more likely to respond to ICIs.

The second study was conducted on three cohorts of patient affected by any stage of RCC [103]. Cohort 1 was a retrospective series of 82 patients, while cohorts 2 and 3 were prospective and enrolled 26 and 92 patients, respectively. In these two prospective cohorts, higher plasma C4d levels had a negative prognostic impact in terms of PFS, albeit not a statistically significant one. A trend between high levels of C4d tissue deposition and increased plasma C4d was observed in cohort 3. Moreover, co-localization of C1q with C1s and C4d with C1s indicated the CP of the CS as the main source of C4d deposition. Furthermore, combining the C4d deposits and C4d plasma values, it was possible to distinguish patients with a poor prognosis (high/high) from those with an intermediate (low/high) and good prognosis (low/low), respectively. Based on immunohistochemical sample examination of cohort 1, patients in the high-C1s group (C1s expressed in >30% of tumor cells) had a significantly decreased OS (*p* = 0.006) and PFS (*p* = 0.004) compared with those in the low-C1s group (C1s expression < 30%). High levels of C1s expression were correlated with TNM and metastatic status and they were associated with a worse prognosis for early disease stages (I and II), but not for advanced RCC (stage III and IV) in the ccRCC TCGA cohort. No correlation was observed between C1s expression and Fuhrman grade. These results were confirmed also in cohorts 1 and 3. Moreover, the prognostic value of C1s was independent regardless of other complement activation markers, indicating the possible cascade-independent role of C1s. C1s is involved also in tumor cell proliferation. In fact, silencing of C1S in Caki-1 and A498 cells induced a decrease in the proliferation capacity. The number of C1s+ cells within the tumor correlated with increased CD8+ T cell infiltration and PD-1 expression as detected using IHC in cohort 1, but this was not observed for C4d deposits or plasma C4d and C3, underling the potentially complement-independent role of C1s in the modulation of the TME [104]. Based on these results, the authors hypothesized a noncanonical intracellular function of C1s. In particular, three nonexclusive processes were suggested: (i) tumor cells secreted C1r, C1s, C3, C4, and C5, which, in association with C1q macrophage production, led to the canonical complement cascade activation; (ii) C1s modified the tumor cell transcription program and phenotype and promoted their proliferation and survival; (iii) a complex synergic interaction between the C1q-producing tumor cells and T cells was observed. In fact, IFNγ stimulated the production of C1s. The C1s-expressing tumor cells failed to activate CD4 and CD8 T cells. In contrast, C1s depletion in the tumor cells resulted in the aberrant expression of MHC class I and alleviated the inability of the tumor cells to activate the CD8 T cells [103]. Therefore, this study suggested that C1s not only took part in the activation of the classical complement pathway cascade but it also promoted tumor cell proliferation and would appear to have a role in conditioning tumor immunogenicity (higher T cell infiltrate and PD-L1 expression). In summary, this second study confirmed the negative prognostic value of the CS and in particular of C1s, in line with what was previously reported by Roumenine et al. Moreover, it evaluated for the first time the complement as a circulating biomarker, showing a correlation between plasmatic CS values and C4d tissue expression [103].

Based on the results of the previous two studies, a mechanism of classical pathway activation of the complement in “aggressive complement” tumors has been proposed (Figure 3). The first step is represented by C1q TAM secretion, which contributes to pro-tumoral TAM phenotypes, T cell exhaustion, the adhesion of tumor cells to the extracellular matrix, and neoangiogenesis. RCC cancer cells produce C1r and C1s, leading to an active C1 complex formation. This, in association with the IgG deposits on the tumor cells, promotes the classical pathway of the CS. Tumor cells also secrete the subsequent complement components, leading to C4 and C3 activation fragment deposition (C4b, C4d, C3b, iC3b, C3d). Moreover, the anaphylatoxins C3a and C5a produced by the activation of the classical pathway modulate the tumor cells and TME [16].

The role of other complement components in ccRCC was explored more deeply. An Asian study conducted on 272 RCC patients who underwent nephrectomy demonstrated the negative prognostic impact of high C5a histochemical expression in term of disease-free survival (DFS) (*p* = 0.079) and OS (*p* = 0.011), with a statistical significance for OS observed only for stages III and IV (*p* < 0.001) [59].

A more recent study confirmed the negative prognostic significance of a high expression of C5a in mRCC. This trial included 231 patients that received a TKI (sorafenib or sunitinib as first-line treatment). All tissue samples analyzed (mainly from nephrectomy and only a minority from metastases) were obtained prior to the start of TKI treatment. High immunohistochemical expression of C5a was significantly correlated with a worse OS and PFS (*p* = 0.0199 and *p* = 0.0138, respectively). C5a expression was significantly correlated with the MMP9 (*p* = 0.000), vimentin (*p* = 0.000), tumor PD-L1 (*p* = 0.001), stromal PD-L1 (*p* = 0.002), PD-1 (*p* = 0.003), and Ki67 (*p* = 0.000) expression levels. Additionally, high levels of C5a expression were strongly correlated with resistance to TKI (*p* < 0.001). Multivariate analysis identified C5a expression as an independent prognostic factor for mRCC patient outcomes [105].

Another study clarified the prognostic role of C3, analyzing three gene expression datasets (GSE36895 contained data from 29 ccRCC tissue samples and 23 tumor adjacent tissue samples; GSE53757 from 72 ccRCC samples and 72 tumor-adjacent samples; GSE66272 from 26 ccRCC samples and 26 tumor-adjacent samples) and a cohort of RCC patients in the TCGA database. Survival analysis was conducted only for this last cohort. In particular, C3, FN1, and C3AR1 were all more highly expressed in the RCC tissues (*n* = 539) than healthy renal tissues (*n* = 72) (*p* < 0.001). However, the protein levels of C3 and FN1, but not of C3AR1, were significantly higher in the RCC tissues than in normal kidney tissues. The RCC patients with high C3 or FN1 expression had a poorer OS (all *p* < 0.05). High C3 expression was also associated with significantly worse relapse-free survival (RFS). C3AR1 had no prognostic value in terms of both OS and RFS [106]. These studies were concordant and supported the negative prognostic significance of the CS in RCC.

The role of FH, which is part of the AP, was also explored in RCC. From histochemical tissue examination, the expression of FH was heterogeneous since it was possible to recognize both extracellular (mb-FH) and intracellular (int-FH) deposits. The co-localization of mb-FH with IgG suggested complement activation. Tumor cells were the main cells staining positive for FH. This study examined four cohorts of patients, three of which included ccRCC patients. Retrospective cohorts 1 and 2 enrolled a total of 224 patients and cohort 4 prospectively analyzed 61 patients. Histological analysis was conducted on cohorts 1 and 2, while cohort 4 was considered for plasma analysis. The Mb-FH deposits did not impact survival, whereas a high int-FH density was significantly correlated with a worse outcome. FH-silenced ccRCC cell lines were characterized by an alteration in proliferation, the cell cycle (arrested at G0–G1), morphology, viability, and migration. Int-FH but not mb-FH seemed to be essential for the tumor cell phenotype. Therefore, int-FH could play a role as a regulator of complement activation and impact the survival outcomes [107].

PTX3 was evaluated as a prognostic and predictive factor in a prospective cohort of 168 RCC patients. Both in cell lines and tissue samples, PTX3 was more highly expressed in cancer cells than in healthy ones. Moreover, PTX3 was co-localized with C1q, CD59, C3aR, and C5R. At time of diagnosis, the PTX3 serum levels were higher in the patients with RCC compared to healthy controls (*p* < 0.001) and significantly correlated with a high Fuhrman grade (G3–4 vs. G1–2 *p* < 0.01), lymph node involvement (N1 vs. N0 *p* = 0.0008), and visceral metastases (M1 vs. M0 *p* < 0.001). Patients with low baseline PTX3 levels (<165.0 pg/mL) showed a significantly higher 10-year OS rate as compared with ccRCC patients with high PTX3 serum levels (73.7% vs. 48.4%, *p* = 0.002) [108].

Thus, all studies examined (Table 2) were concordat on the negative prognostic value of different components of the CS, although a prospective, large, and more homogeneous cohort of patients is required for their validation, and further analyses are highly expected for assessing their potential role as predictors of response at the immunotherapy stage.

## 5. The Complement System as a Possible Therapeutic Target

Rees et al. measured the plasma serum levels of some complement components, including C1q, C3, C5, FB, FD, FH, FI, C3c, sCD59, and s5b-9 (terminal complement complex—TCC) in a prospective sample of 24 mRCC patients prior to the initiation of immunotherapy (nivolumab monotherapy or a combination of ipilimumab and nivolumab).The concentration of plasma complement proteins was correlated with the time to next treatment (TNT). Patients with FH and FD levels below the cutoff had a worse response to ICIs, while low levels of both FI and TCC were associated with a better response and longer TNT. The authors also proposed an algorithm in order to select ICI-sensitive patients: low levels of TCC were predictive of the ICI response. In the presence of high TCC values, high C5 levels determined a good response to ICIs [17]. This study also evaluated a murine RCC model resistant to ICIs. Mice treated with C3aR1 (SB290157) and C5aR1 (PMX53) inhibitors had reduced tumor growth. The correlation between tumor growth and complement activation was not evaluated. Genetic deficiencies and pharmacological blockade of the complement receptors improved TIL function (increased production of IFN-γ). In conclusion, the complement appears to act as an additional checkpoint in RCC [17]. Although this prospective study enrolled limited series of mRCC patients and did not lead to robust conclusions, it is of great interest and suggests further prospective investigation to better clarify the predictive role of the CS in immunotherapy so as to guide treatment selection in the near future.

Another preclinical study evaluated a double block of both C5a and PD-1 in a syngeneic mouse model based on the subcutaneous growth of 393P cells that constitute a KRAS-driven lung adenocarcinoma. The combination of anti-C5a and anti-PD-1 monoclonal antibodies resulted in a significant reduction in tumor growth, showing the synergistic effect of the combined inhibition of both immune checkpoints and the complement, warranting additional studies for evaluating this potential synergistic therapeutic strategy [18].

## 6. Discussion

Our review further explored the central role of the CS and in particular the activation of the CP in RCC. Chronic imbalanced activation of the CS (especially the CP) negatively modulates the activity of the immune system, leading to an immunosuppressed TME. This favors tumor growth and progression [109]. A mechanism of complement activity proposed in RCC considers the C1q-secreting TAMs central in the first step of intra-tumor complement activation [16,102]. Then, the complex interaction between the tumor cells secreting the other components of the complement (C1r, C1s, C3, and C4), the MDSCs, and exhausted T lymphocytes leads to proliferation, increased tumor invasiveness, and neoangiogenesis, which ultimately results in tumor progression. In addition, there is new evidence regarding non-canonical mechanisms that involve the CS and that make the scenario even more complex [102,103,104,107].

All the studies reported in our review, although retrospective, agree on the negative prognostic role of the CS components’ expression. Specifically, high levels of complement factors, tested in both tissue and plasma, are correlated with a worse outcome in RCC patients, mainly in the advanced stages (III–IV) [59,102,103,106,107,108]. However, these data need be validated prospectively.

Furthermore, the role of the CS as a predictor of the response to ICIs is of great interest, warranting further investigation. The only prospective study that evaluated the predictive role of the CS involved a limited number of patients with mRCC (24 total) but could be a first exploratory study for the realization of a prospective clinical validation trial. In fact, this study identified 11 plasma-detectable complement proteins (C1q, C3, C5, FB, FD, FH, FI, C3c, sCD59, and s5b-9), which were quantified prior to treatment with ICIs (nivolumab or ipilimumab/nivolumab). The study hypothesized an algorithm for selecting those patients more likely to respond to ICIs based on the plasma values of TCC and C5a [17]. The above findings show that the complement could play a crucial role in determining an immunosuppressed TME.

Therefore, high levels of CS components could identify highly immunosuppressed tumors, which therefore could be more responsive to ICIs. In this setting, ICIs would restore the proper activity of the immune system against cancer cells. This evidence demonstrates that high complement levels could be predictive of ICI response and this could be used as a positive predictive factor in clinical practice. Indeed, as already stated before, several studies have demonstrated a correlation between high levels of complement factors and the expression of molecules such as PD-L1 and LAG3. This is representative of an exhausted TME, which is therefore more susceptible to the action of ICIs [102]. In fact, exhausted CD8+ TILs with a mild expression of PD-1 could be re-activated by a PD-1/PD-L1 blockade [110,111,112]. However, the role of the CS in the development of treatment resistance to ICIs in mRCC has yet to be clarified, as there are conflicting data. Probably, the complex interaction between the TME and tumor cells has a fundamental role in the response to immunotherapy and anti-angiogenic agents [27,113].

It is important to notice that most of these studies evaluated only complement activation in tumor tissue. It would be interesting to evaluate the role of the CS as a dynamic biomarker of response/resistance in the course of immunotherapy by evaluating plasma levels. In fact, having a dynamic marker that could be easily evaluated in plasma during systemic therapy could be fundamental in selecting those patients who would benefit most from immunotherapy rather than anti-VEGFR-based treatments.

The CS represents only one of the pathways studied in order to identify new prognostic and predictive biomarkers in mRCC patients. In recent years, given the advent of ICI-based combinations as a first-line therapy for mRCC, the research has focused on the definition of biological features, including new immune-modulatory and angiogenesis gene expression signatures (GESs) that could play a role as predictors of response to guide clinicians in the selection of the most active combination. Therefore, exploratory analyses from four main randomized prospective trials (JAVELIN Renal 101, IMmotion151, IMmotion150, and CheckMate 9ER) of ICI-based combinations in treatment-naïve mRCC patients tried to identify potential biomarkers of response, however, without any definitive conclusion. Motzer et al. investigated GESs in histological samples from the phase 3 JAVELIN Renal 101 trial (evaluating avelumab plus axitinib as a first-line therapy for mRCC patients compared to sunitinib monotherapy). This analysis demonstrated that the expression of immune signatures, which included natural-killer-cell-related transcripts, as well as chemokine- and cytotoxic-T-cell-related elements, was associated with an improved PFS with ICI-based treatment compared to anti-VEGFR monotherapy [114]. Similarly, an exploratory analysis of integrated RNA sequencing (RNA-seq) and targeted somatic variant analysis from tumor samples of the IMmotion151 study (comparing atezolizumab plus bevacizumab to sunitinib) defined seven clusters of RCC associated with a different response to ICI-based combinations and VEGFR-TKI alone. The T-effector/proliferative cluster (number 4), proliferative cluster (number 5), and snoRNA cluster (number 7) responded better to immunotherapy. Moreover, regardless of the clusters, among the patients responding to immunotherapy, genes associated with the proliferation and immune pathways were more expressed. On the other hand, the patients responding to sunitinib showed a major expression of genes associated with VEGF signaling (hypoxia) [9]. A translational exploratory analysis from IMmotion150 was conducted with the aim of defining molecular biomarkers potentially correlated with the clinical outcome in each treatment group (atezolizumab vs. atezolizumab + bevacizumab vs. sunitinib). This analysis indicated that tumor mutation and neoantigen burdens were not associated with PFS, whereas angiogenesis, the T-effector/IFN-γ response, and myeloid inflammatory gene expression signatures were strongly and differentially associated with PFS within and across the treatments [10]. During the 2023 ASCO Genitourinary Symposium, Choueiri presented the results of a post hoc molecular analysis on pre-treatment tumor samples from the CheckMate 9ER trial. The study compared the combination of nivolumab and cabozantinib with sunitinib in a first-line setting for mRCC patients. The analysis identified a high angio-GES was associated with a longer PFS compared to a medium and low angio-GES in patients receiving nivolumab + cabozantinib; however, the predictive value of all seven GESs was not confirmed using Cox PH models. In addition, no single gene with predictive value was identified [8].

Further prospective studies are needed to confirm and deepen the role of the CS in RCC. It would be interesting, based on the study of Reese et al. [17], to design clinical studies aimed at evaluating the efficacy of combining ICIs with a complement inhibitor.

## 7. Conclusions

In actuality, the treatment selection in first-line mRCC therapy is currently based on the IMDC’s prognostic factors, which rely only on clinical data and do not reflect the molecular heterogeneity of RCC tumors. Preliminary gene sequencing studies have paved the way for the identification of those tumors more sensitive to ICIs rather than to anti-VEGFR agents. However, further studies are highly expected to identify prognostic and predictive factors that can also be applied in clinical practice. In particular, the goal would be to identify an easily detectable, reproducible, and highly specific molecular profile able to select the best treatment option for each patient for a tailored personalized treatment approach. In fact, identifying patients who could benefit from immunotherapy alone could lead to more personalized and less toxic treatment strategies, such as the possibility of intermittent use of VEGFR-TKIs while maintaining immunotherapy in those patients with a disease response after initial induction with the ICI-TKI combo [115].

In conclusion, the CS, in consideration of its role in modulating immunogenicity and angiogenesis in RCC, could represent a dynamic marker with a predictive value of the response to ICI-based therapies. Further prospective investigations are highly awaited.

## Figures and Tables

**Table 1:** The relationship between the complement and coagulation systems.

Factor	Substrate	Action of the Complement/Coagulation Factor on the Substrate: Activated (+)/Inactivated (−)	References
Factor XIIa	C1r	+	[37]
Factor XIII, Fibrinogen, Fibrin, Thrombin	MASP1	+	[38,40,42,43]
Fibrin, Thrombin	MASP2	+	[43,46,47]
Thrombin, Factor VII, Factor IXa, Factor Xa, Factor Xia, Kallikrein, Plasmin	C3 and C5	+	[40,48,49,50,51,52,53]
Platelets	C3	+	[50,51,54,55]
Thrombin	DAF	+ (− complement activity)	[41]
MAC, C5a	Tissue Factor	+	[56,57]
Complement	Heparin	-	[58]

**Table 2:** Negative prognostic components of the complement system investigated in RCC.

Study	Negative Prognostic Complement Factor Evaluated	Laboratory Investigation	Patients (*n*)	Type of Study [R: Retrospective; P: Prospective]	Stage (%)	Survival Outcome
L.T. Roumenina et al., Cancer Immunol. Res., 2019 [102]	C1q C3 C4 C4d	IHC	Cohort 1: 106	R	I (40) II (6) III (41) IV (14)	**C1q** **OP: PFS *p* = 0.008, OS *p* = 0.0016** Stage I–II: PFS *p* = 0.711, OS *p* = 0.256 **Stage III–IV: PFS *p* = 0.00356, OS *p* = 0.00198**
						**C4c OP: PFS *p* = 0.0235, OS *p* = 0.0299**
						**C4d OP: PFS *p* = 0.013, OS *p* = 0.007**
						**C3 OP: PFS *p* = 0.0349, OS *p* = 0.07**
			Cohort 2: 154	R	I (40) II (5) III (54) IV (2)	**C1q:** Stage I–II: PFS *p* = 0.527, **Stage III–IV: PFS *p* = 0.0109**
			Cohort 3: 43	R	IV (100)	**C1q: PFS *p* = 0.00276, OS *p* = 0.00126** **C4d: PFS *p* = 0.0176**
M.V. Daugan et al. Cancer Immunol. Res., 2021 [103]	C1s C4d deposits Plasma C4d	IHC IHC Plasma	Cohort 1: 82	R	I (40) II (6) III (39) IV (15)	**C4d deposits OP: PFS *p* = 0.00176**
			Cohort 2: 26	P	/	Plasma C4d OP: PFS *p* = 0.09
			Cohort 3:92 (longer FU)	P	I (54) II (8) III (17) IV (18)	**Plasma C4d OP: PFS *p* = 0.0125**
Wei Xi et al. Scientific Reports, 2016 [59]	C5a	IHC	272	R	I (62) II (8) III (24) IV (7)	OP: **OS *p* = 0.011**, DFS *p* = 0.079 Stage I–II: OS *p* = 0.845 Stage III–IV: OS *p* < 0.001
C. Yang et al. IJBM, 2023 [105]	C5a	IHC	231		IV	**OS *p* = 0.0199, PFS *p* = 0.0138**
Dong et al., BMC 2021 [106]	C3 C3AR1	Transcriptomics analysis	532 (TCGA-KIRC dataset)	R		C3 OP: OS *p* = 0.0004, RFS *p* = 0.007 C3AR1 OP: OS *p* = 0.204, RFS *p* = 0.323
Daugan et al. Cancer Immunol. Res., 2021 [107]	mb-FH int-FH	IHC	Cohort 1: 133	R	I (0) II (24) III (64) IV (9)	OP mb-FH: DFS *p* = 0.14 **OP int-FH: DFS *p* = 0.004**
			Cohort 2:91	R	I (5) II (22) III (48) IV (8)	OP mb-FH: PFS *p* = 0.226, OS *p* = 0.627 **OP int-FH: PFS *p* = 0.0274, OS *p* = 0.0727**
Netti et al. Aging (Albany NY). 2020 [108]	PTX3	Plasma	Cohort 1: 168	R	pT1 (62) pT2 (14) pT3 (21) pT4 (3) pN+ (20) cM+ (18)	**10-yr OS rate: 73.7% ↓PTX3 vs. 48.4% ↑PTX3, *p* = 0.002**

Abbreviations: IHC, immunohistochemistry; OP, overall population; OS, overall survival; PFS, progression-free survival; C4d, C4 deposits: RFS, relapse-free survival; DFS, disease-free survival. Up arrows: “high level”; down arrows: “low level”.
